# Signatures of electronic and nuclear coherences in ultrafast molecular x-ray and electron diffraction

**DOI:** 10.1063/4.0000043

**Published:** 2021-01-11

**Authors:** Jérémy R. Rouxel, Daniel Keefer, Shaul Mukamel

**Affiliations:** Department of Chemistry and of Physics and Astronomy, University of California, Irvine, California 92697-2025, USA

## Abstract

Femtosecond x-ray and electron diffraction hold promise to image the evolving structures of single molecules. We present a unified quantum-electrodynamical formulation of diffraction signals, based on the exact many-body nuclear + electronic wavefunction that can be extracted from quantum chemistry simulations. This gives a framework for analyzing various approximate molecular dynamics simulations. We show that the complete description of ultrafast diffraction signals contains interesting contributions involving mixed elastic and inelastic scattered photons that are usually masked by other larger contributions and are neglected. These terms include overlaps of nuclear wavepackets between different electronic states that provide an electronic decoherence mechanism and are important for the time-resolved imaging of conical intersections.

## I. INTRODUCTION

Over the past century, x-ray and electron diffraction signals have become the main tool for exploring the structure of matter.^2^ The stationary x-ray diffraction (XRD) pattern in crystals is given by the square of the Fourier transform of the ground state electronic charge density. Electrons, in contrast, are diffracted from the total (electronic + nuclear) charge density. Diffraction is usually dominated by the 2-molecule (or more generally 2-scatterer) coherent response that is responsible for the formation of Bragg peaks. For single molecule diffraction or diffraction in the absence of order, e.g., from liquids, the structure factor vanishes due to the absence of a long-range order and a continuous pattern is observed in momentum space.^21,28^ In contrast to the 2-scatterer case, this 1-molecule diffraction pattern may not be written as a modulus square of a scattering amplitude.^6^

Stationary x-ray and electron diffraction signals directly probe the charge densities. The advent of x-ray Free Electron Lasers (XFELs) has permitted the measurement of diffraction patterns from nanocrystals and time-evolving structures^8,13^ with subfemtosecond resolution.^7,9,12,22,23,34^ It is tempting to assume that for a time-evolving charge density, these signals simply give instantaneous snapshots of the charge density. This picture is, however, incomplete and the signals depend on coherences and inelastic scattering that go beyond the charge density.^26^ For an excited molecule prepared in a time-evolving superposition of many-electron states, the signal may not be expressed in terms of the instantaneous charge density alone.

Here, we derive general expressions for diffraction signals in molecules that are ready to employ *ab initio* electronic structure calculations of nonadiabatic dynamics in the joint electronic + nuclear space. Specific signatures of conical intersections are identified. A correct description of the signals requires treating the charge density as an operator and taking into account its different matrix elements. Since the diagonal matrix elements scale as the number of electrons in the system and off diagonal elements have contributions from a few electrons involved in the relevant electronic transitions, the ultrafast XRD or UED (ultrafast electron diffraction) signals are dominated by the former. The off diagonal elements that contribute to inelastic signals are thus often neglected. The independent atoms model (IAM) is widely used to describe the diffraction signals,^17,24^ which assumes that each atom contributes to the signal with its own structure factor. This approximation fails to describe valence electrons involved in chemical bonding. This is justified for stationary diffraction but misses bond forming and breaking. *Ab initio* calculations are widely used to recover the experimental XRD and UED signals.^30,32,33^ There exist different approximations for the XRD and UED signals, owing to the different levels of treatment of the molecular wavefunction. Phenomenological descriptions are often sufficient to reproduce specific experiments, but they miss certain features of the diffraction signal.

We present a rigorous formulation of time-resolved diffraction signals in the joint electronic + nuclear space that contains all contributions to the signals. Contributions that mix diagonal and off diagonal matrix elements of the charge density are highlighted. These involve nuclear wavepackets in different electronic surfaces and are of importance in the study of decoherence effects in conical intersection. The electronic and nuclear charge densities are single-body operators in their respective subspaces but many-body effects enter through the many-body wavefunction used to compute their matrix elements. We show that such contributions can in principle be extracted by the separate detection of elastic, inelastic, and non-frequency resolved terms. Covariance techniques that make use of the stochastic properties of FEL SASE light can be adopted to that end.^3^

Electron diffraction probes the total charge density.^10^ Interestingly, the terms often missed in XRD (mixed terms of diagonal and off diagonal electronic charge densities) also enter in UED through mixed nuclear-electronic charge densities (diagonal nuclear and off diagonal electronic densities).

## II. THE ELECTRONIC CHARGE DENSITY OPERATOR

The electronic charge density is a single body operator given by

$$\sigma^{E}(r)=\sum_{i}^{n}e\delta (r-r_{i})=\sum_{i}^{n}e|r_{i}\rangle \langle r_{i}|=e\psi^{†}(r)\psi (r),$$ (1)

where *n* is the number of electrons and $\psi^{†}(r\prime )$ and ψ(r′) are the fermionic field creation and annihilation operators expanded in a single-electron basis $\phi_{\alpha }$,

$$\psi^{†}(r)=\sum_{\alpha }\phi_{\alpha }^{*}(r)c_{\alpha }^{†},$$ (2)

$$\psi (r)=\sum_{\alpha }\phi_{\alpha }(r)c_{\alpha }.$$ (3)

Substituting Eqs. (2) and (3) in Eq. (1) gives

$$\sigma^{E}(r)=\sum_{\alpha \beta }\phi_{\alpha }^{*}(r)\phi_{\beta }(r)c_{\alpha }^{†}c_{\beta }.$$ (4)

This expression can be recast as

$$\sigma^{E}(r)=e\gamma^{\left(1\right)}(r,r),$$ (5)

where $\gamma^{\left(1\right)}(r,r\prime )=\psi^{†}(r\prime )\psi (r)$ is the one-electron density operator.

X-ray diffraction measures the Fourier transform of the charge density operator

$$\sigma^{E}(q)=e\sum_{i}^{n}e^{-iq\cdot r_{i}}=e\sum_{k}c_{k}^{†}c_{k+q},$$ (6)

where ***q*** is the momentum transfer vector. XRD carried out on a single molecule measures the expectation value of the product of two charge density operators

$$\sigma^{E}(-q)\sigma^{E}(q)=e^{2}\sum_{k,k\prime }c_{k}^{†}c_{k-q}c_{k^{\prime }}^{†}c_{k\prime +q}.$$ (7)

In real space, this gives

$$\begin{array}{c} \sigma^{E}(r)\sigma^{E}(r\prime )=e^{2}\psi^{†}(r)\psi (r)\psi^{†}(r\prime )\psi (r\prime )\\ =\gamma^{\left(2\right)}(r\prime ,r;r\prime r)+\gamma^{\left(1\right)}(r,r)\delta (r-r\prime ) \end{array},$$ (8)

where $\gamma^{\left(2\right)}(r_{1},r_{2},r_{1}^{\prime },r_{2}^{\prime })=\psi^{†}(r_{2}^{\prime })\psi^{†}(r_{1}^{\prime })\psi (r_{1})\psi (r_{2})$ is the two-electron density operator.

## III. THE X-RAY DIFFRACTION SIGNAL FROM A SINGLE MOLECULE

The XRD signal is defined in Appendix A as the integrated rate of photon number change during the scattering process. Starting from Eq. (A5) and assuming a very short (impulsive) x-ray pulse, the single molecule x-ray diffraction signal can be recast as

$$S_{\text{XRD}}(q,T)\propto ℜ\langle \Psi (T)|\sigma^{E}(-q)\sigma^{E}(q)|\Psi (T)\rangle ,$$ (9)

where |Ψ(T)⟩ is the molecular many-body wavefunction in the joint electronic and nuclear space. Note that although $\sigma^{E}(r)$ is a single body electronic operator, its expectation value is calculated in the joint nuclei + electrons space and depends on nuclear many-body wavefunction overlaps. In the following, we expand |Ψ(T)⟩ in the adiabatic basis set consisting of products of nuclear and electronic wavefunctions

$$\left|\Psi (T)\rangle =\sum_{i}|\chi_{i}(T)\rangle |\varphi_{i}\right\rangle ,$$ (10)

where $\left|\varphi_{i}\right\rangle$ are the many-body electronic states which may depend parametrically on nuclear coordinates and $\left|\chi_{i}(T)\right\rangle$ are the nuclear many-body wavefunction in the state *i*. $\left|\chi_{i}(T)\right\rangle$ in Eq. (10) are not normalized and include the amplitude in-state *i*. Alternatively, normalized nuclear wavefunctions $c_{i}(T)|{\tilde{\chi }}_{i}(T)\rangle =|\chi_{i}(T)\rangle$ can be introduced, where $c_{i}(T)$ is the amplitude in-state *i*. This wavefunction acts in the |r,R⟩ space where ***r*** and ***R*** are the electronic and nuclear coordinates, respectively,

$$\left\langle r,R|\Psi (T)\rangle =\sum_{i}\chi_{i}(R,T)\varphi_{i}(r,R\right).$$ (11)

The adiabatic basis set provides a convenient representation of the exact molecular wavefunction; note that we are not making the adiabatic approximation. Electronic structure simulations are usually performed in the adiabatic basis under the Born–Oppenheimer approximation, which states that the electron and nuclear motions can be separated due to their different timescales. The resulting adiabatic electronic states $\varphi_{i}(r,R)$ depend parametrically on the nuclear configuration. Exact quantum dynamical simulations can then be performed on these surfaces by including non-adiabatic couplings that account for conical intersection regions, where the motion of electrons and nuclei becomes strongly coupled and non-Born-Oppenheimer effects kick in. This yields a time-dependent nuclear wavepacket $\chi_{i}(R,T)$ in each adiabatic state, and the exact many-body wavefunction |Ψ(T)⟩, Eq. (10). Other basis sets can be chosen, e.g., the diabatic or exact factorization of the molecular wavefunction.^1^ This affects the wavefunction and the formulation of the diffraction signal, but the terms and contributions discussed here are incorporated there in a similar fashion. Different techniques and approximations have been developed to describe the non-adiabatic dynamics in large molecules. These include surface hopping,^4,25,29^ frozen Gaussian approaches of various types,^5,18,31^ or the multi-configurational time-dependent Hartree.^20^ These approximations of the molecular wavefunction miss certain contributions to the diffraction signal. The present formulation is based on the exact electronic and nuclear wavefunction, and thus a offers a benchmark for other approximate diffraction simulations.

**FIG. 1.**
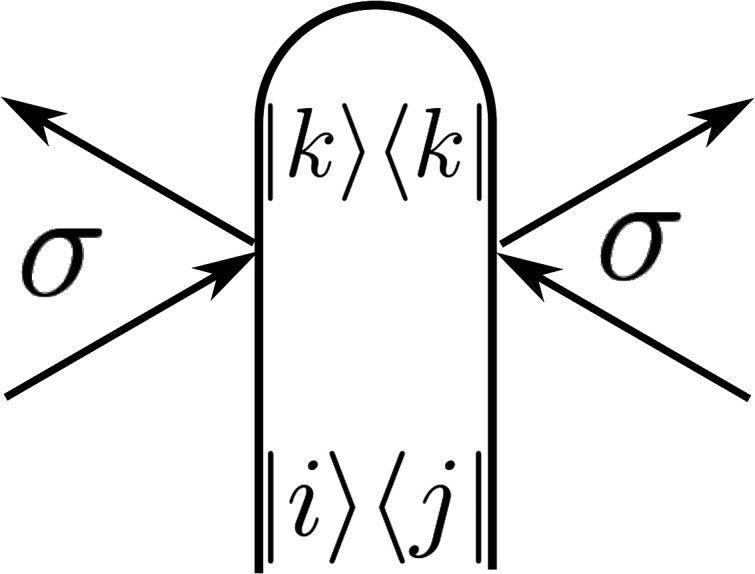
Loop diagram for x-ray diffraction, Eq. (12).

Inserting the many-body wavefunction, Eq. (10), into Eq. (9) yields the various contributions to the 1 molecule XRD signal depicted in Fig. 1,

$$S_{\text{XRD}}(q,T)\propto ℜ\sum_{\mathrm{ijk}}\left\langle \chi_{i}(T)|\sigma_{ik}^{E}(-q)\sigma_{kj}^{E}(q)|\chi_{j}(T)\right\rangle .$$ (12)

When *i* =* k* or *k* =* j*, the matrix elements *σ_ik_* or *σ_kj_* are the ordinary electronic charge densities that involve all the electrons. On the other hand, when i≠k, the matrix elements represent *transition charge densities* that only involve the orbitals involved in a particular transition. As such, they are more sensitive to charge migration between the ground and valence excited states that typically only involve a few electrons.

The various contributions to the signal are conveniently represented by loop diagrams that represent the time evolution of the bra and ket of the molecular many-body wavefunction and the perturbative interactions with the incoming x-ray or electron beams.^19^ If the electronic wavefunction is independent of the nuclear coordinates (the crude-adiabatic basis^16^), then the electronic operators in Eq. (12) can be factorized into electronic and nuclear parts and the signal then depends on an overlap of the nuclear wavepackets

$$S_{\text{XRD}}(q,T)\propto ℜ\sum_{\mathrm{ijk}}\sigma_{ik}^{E}(-q)\sigma_{kj}^{E}(q)\langle \chi_{i}(T)|\chi_{j}(T)\rangle .$$ (13)

For a two electronic state model with a ground state *g* and an excited state *e*, Eq. (12) gives

$$\begin{array}{c} S_{\text{XRD}}(q,T)\propto ℜ(\langle \chi_{g}(T)|\sigma_{gg}^{E}(-q)\sigma_{gg}^{E}(q)|\chi_{g}(T)\rangle \\ +\;\langle \chi_{e}(T)|\sigma_{ee}^{E}(-q)\sigma_{ee}^{E}(q)|\chi_{e}(T)\rangle \\ +\;\langle \chi_{g}(T)|\sigma_{eg}^{E}(-q)\sigma_{eg}^{E}(q)|\chi_{g}(T)\rangle \\ +\;\langle \chi_{e}(T)|\sigma_{ge}^{E}(-q)\sigma_{ge}^{E}(q)|\chi_{e}(T)\rangle \\ +\;2ℜ\langle \chi_{g}(T)|\sigma_{eg}^{E}(-q)\sigma_{ee}^{E}(q)|\chi_{e}(T)\rangle \\ +\;2ℜ\langle \chi_{e}(T)|\sigma_{ge}^{E}(-q)\sigma_{gg}^{E}(q)|\chi_{g}(T)\rangle ) \end{array}.$$ (14)

**FIG. 2.**
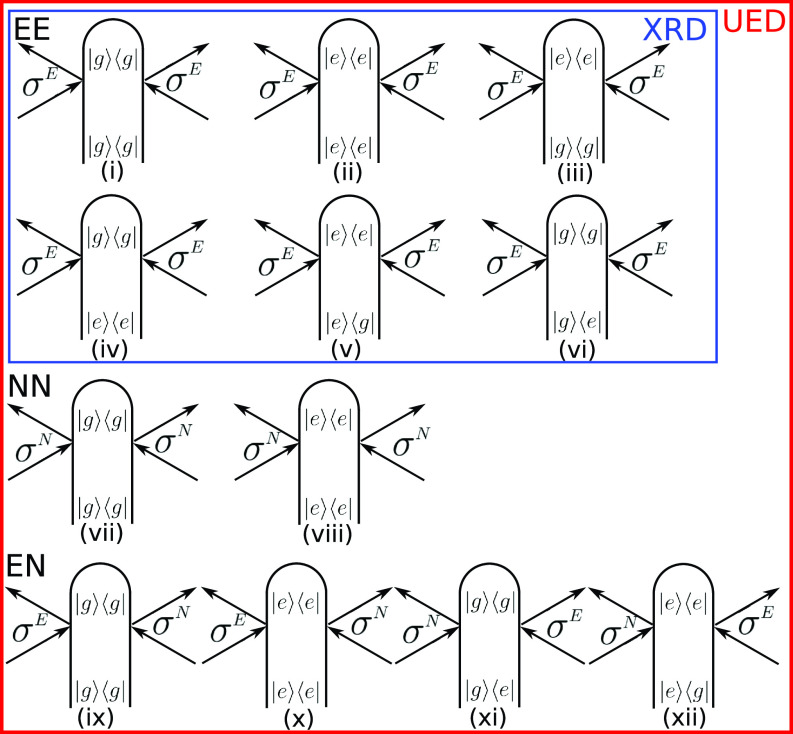
Loop diagrams for one-molecule XRD [Eq. (14)] and UED [Eq. (25)] expanded in electronic eigenstates for a two-state model. XRD is given by diagrams (i)–(vi), while all diagrams contribute to UED. The total XRD signal is the sum of electronic elastic [(i) and (ii)], inelastic electronic Stokes and anti-Stokes [(iii) and (iv)], and mixed electronic elastic/inelastic terms [(v) and (vi)]. UED has additional contributions from nuclear elastic [(vii) and (viii)], the mixed nuclear/electronic elastic [(ix) and (x)], and mixed nuclear/electronic elastic/inelastic [(xi) and (xii)] scattering.

The first two terms are the elastic contributions from the ground and excited states, whereas the following two terms represent inelastic Stokes and anti-Stokes processes. Finally, the last two terms represent mixed elastic/inelastic contributions. As can be read from the last two diagrams in Fig. 2, the mixed terms involve an elastic scattering involving *σ_gg_* or *σ_ee_* and an inelastic one with *σ_eg_* or *σ_ge_*. The two scattered photon amplitudes have frequencies centered around *ω_X_* and $\omega_{X}\;\pm \;\omega_{eg}$, where *ω_X_* is the incoming x-ray frequency and *ω_eg_* is the transition frequency between states *e* and *g*. In order to generate a signal, a population must be created on the detector, which is possible only if these two amplitudes have a frequency within the detector bandwidth. The mixed terms can be observed only by a broadband detector with a bandwidth larger than *ω_eg_*. This is usually the case for XRD detectors since in most cases $\omega_{eg}\ll \omega_{X}$. This quantity depends on the molecular properties, i.e., the energy splitting of the involved electronic states. The latter is usually larger in the Franck–Condon region (between one and several eV), and smaller between higher excited states or in the vicinity of conical intersections.

XRD signals are usually computed either within the Independent Atom Model (IAM) or by *ab initio* techniques. The IAM completely misses the motion of valence electrons (bond forming and breaking).^24^ Even with *ab initio* calculations, the last two contributions in Eq. (14) are often not included in the analysis of experimental data^11,17^ but have been pointed out in former studies.^6,14,15,27^ These are prominent at conical intersections, where the overlap of nuclear wavepackets provides a decoherence mechanism, and thus carries valuable information about the non-adiabatic passage.

## IV. TWO-MOLECULE X-RAY DIFFRACTION SIGNALS

We now repeat the above discussion for the 2-molecule coherent diffraction. This signal arises from orientationally ordered molecular assemblies such as crystals. In contrast to the one-molecule signal [Eq. (14)] described by two-point correlation functions ⟨ψ(T)|σσ|ψ(T)⟩, the two-molecule one does not contain products of the density operators, but absolute squares of single operator contributions.^6,19^ This stems from the fact that the interaction with each of the two charge densities occurs on different molecules and the two-point correlation function can be factorized into a product of two single point ones. The signal, Eq. (A5), can then be recast as

$$S_{\text{XRD}}(q,T)\propto |\langle \sigma^{E}(q,T)\rangle {|}^{2}.$$ (15)

By using the expansion of Eq. (10), we obtain

$$S_{\text{XRD}}(q,T)\propto |\sum_{ij}\left\langle \chi_{i}(T)|\sigma_{ij}^{E}(q)|\chi_{j}(T)\right\rangle {|}^{2}.$$ (16)

**FIG. 3.**
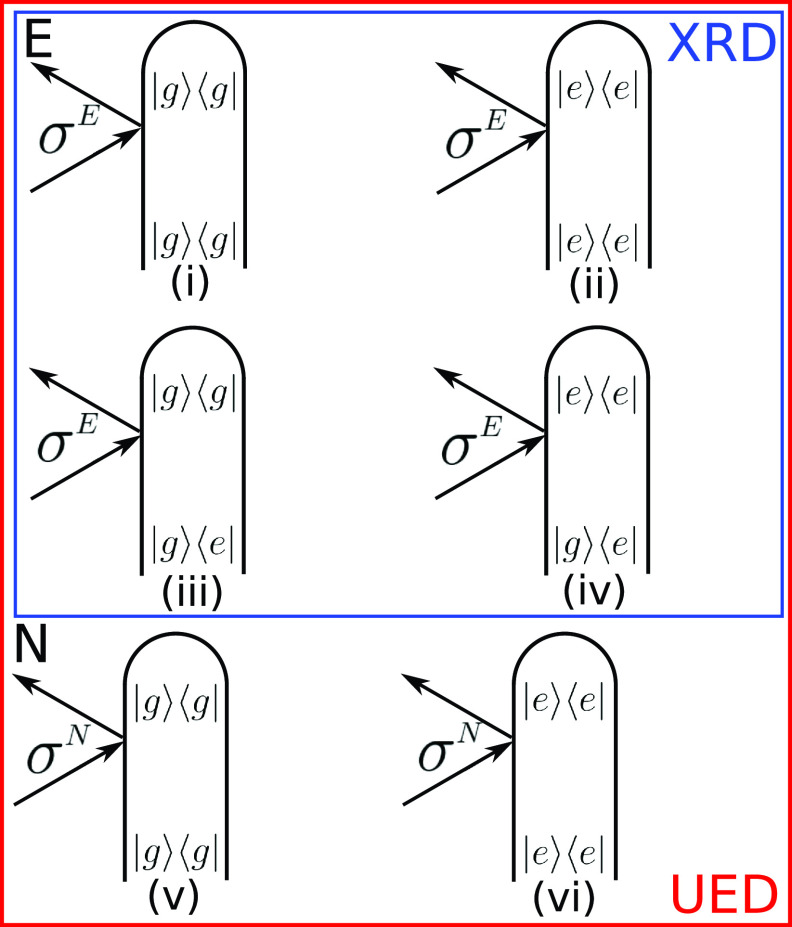
Loop diagrams for two-molecule XRD [Eq. (17)] and UED [Eq. (24)] signals expanded in electronic eigenstates. Diagrams (i)–(iv) contribute to XRD, while all diagrams contribute to UED. The total XRD signal is the sum of electronic elastic [(i) and (ii)] and inelastic electronic Stokes and anti-Stokes [(iii) and (iv)] amplitudes. UED has additional nuclear elastic contributions [(v) and (vi)].

For a two electronic states model, see Fig. 3, this gives

$$\begin{array}{c} S_{\text{XRD}}(q,T)\propto |\langle \chi_{g}(T)|\sigma_{gg}^{E}(q)|\chi_{g}(T)\rangle +\langle \chi_{e}(T)|\sigma_{ee}^{E}(q)|\chi_{e}(T)\rangle \\ +\;\langle \chi_{e}(T)|\sigma_{eg}^{E}(q)|\chi_{g}(T)\rangle +\langle \chi_{g}(T)|\sigma_{ge}^{E}(q)|\chi_{e}(T)\rangle {|}^{2} \end{array}.$$ (17)

The first two terms represent elastic scattering from states *g* and *e*, and the remaining two terms are the Stokes and anti-Stokes which represent inelastic scattering. When the detection has no frequency resolution, the last two terms become complex-conjugates and can be combined.

Expanding the square in Eq. (17), we get an expression similar to Eq. (14),

$$\begin{array}{c} S_{\text{XRD}}(q,T)\propto ({\left\langle \chi_{g}(T)|\sigma_{gg}^{E}(q)|\chi_{g}(T)\right\rangle }^{2}+{\left\langle \chi_{e}(T)|\sigma_{ee}^{E}(q)|\chi_{e}(T)\right\rangle }^{2}\\ +\;{\left\langle \chi_{e}(T)|\sigma_{eg}^{E}(q)|\chi_{g}(T)\right\rangle }^{2}+{\left\langle \chi_{g}(T)|\sigma_{ge}^{E}(q)|\chi_{e}(T)\right\rangle }^{2}\\ +\;2ℜ(\langle \chi_{g}(T)|\sigma_{gg}^{E}(q)|\chi_{g}(T)\rangle \langle \chi_{e}(T)|\sigma_{ee}^{E}(q)|\chi_{e}(T)\rangle )\\ +\;2ℜ\langle \chi_{g}(T)|\sigma_{gg}^{E}(q)|\chi_{g}(T)\rangle \langle \chi_{e}(T)|\sigma_{eg}^{E}(q)|\chi_{g}(T)\rangle \\ +\;2ℜ\langle \chi_{e}(T)|\sigma_{ee}^{E}(q)|\chi_{e}(T)\rangle \langle \chi_{e}(T)|\sigma_{eg}^{E}(q)|\chi_{g}(T)\rangle )\\ +\;2ℜ\langle \chi_{g}(T)|\sigma_{gg}^{E}(q)|\chi_{g}(T)\rangle \langle \chi_{g}(T)|\sigma_{ge}^{E}(q)|\chi_{e}(T)\rangle \\ +\;2ℜ\langle \chi_{e}(T)|\sigma_{ee}^{E}(q)|\chi_{e}(T)\rangle \langle \chi_{g}(T)|\sigma_{ge}^{E}(q)|\chi_{e}(T)\rangle ). \end{array}$$ (18)

In the absence of frequency resolution, the last two terms in Eq. (17) can be combined and we get

$$\begin{array}{c} S_{\text{XRD}}(q,T)\propto ({\left\langle \chi_{g}(T)|\sigma_{gg}^{E}(q)|\chi_{g}(T)\right\rangle }^{2}+{\left\langle \chi_{e}(T)|\sigma_{ee}^{E}(q)|\chi_{e}(T)\right\rangle }^{2}\\ +\;4ℜ{\left\langle \chi_{e}(T)|\sigma_{eg}^{E}(q)|\chi_{g}(T)\right\rangle }^{2}\\ +\;2ℜ(\langle \chi_{g}(T)|\sigma_{gg}^{E}(q)|\chi_{g}(T)\rangle \langle \chi_{e}(T)|\sigma_{ee}^{E}(q)|\chi_{e}(T)\rangle )\\ +\;4ℜ\langle \chi_{g}(T)|\sigma_{gg}^{E}(q)|\chi_{g}(T)\rangle ℜ\langle \chi_{e}(T)|\sigma_{eg}^{E}(q)|\chi_{g}(T)\rangle \\ +\;4ℜ\langle \chi_{e}(T)|\sigma_{ee}^{E}(q)|\chi_{e}(T)\rangle ℜ\langle \chi_{e}(T)|\sigma_{eg}^{E}(q)|\chi_{g}(T)\rangle ) \end{array}.$$ (19)

## V. ULTRAFAST ELECTRON DIFFRACTION

UED is described by similar expressions to XRD with the substitution

$$\sigma^{E}(q)\to \frac{\sigma^{T}(q)}{q^{2}},$$ (20)

where

$$\sigma^{T}(r)=\sigma_{E}(r)+\sigma_{N}(r),$$ (21)

$$\sigma^{N}(r)=\sum_{I}eZ_{I}|R_{I}\rangle \langle R_{I}{|}_{R_{I}=r},$$ (22)

and *I* labels the nuclei [*i* was used for the electrons in Eq. (1)]. We assume *n* electrons and *N* nuclei. The 1 molecule and 2 molecule UED signals are given by

$$S_{\text{UED}}^{1\text{mol}}(q,T)\propto \frac{1}{q^{4}}ℜ\langle \Psi (T)|\sigma^{T}(-q)\sigma^{T}(q)|\Psi (T)\rangle ,$$ (23)

$$S_{\text{UED}}^{2\text{mol}}(q,T)\propto \frac{1}{q^{4}}|\sum_{ij}\left\langle \chi_{i}(T)|\sigma_{ij}^{T}(q)|\chi_{j}(T)\right\rangle {|}^{2}.$$ (24)

For a two electronic state model, Eq. (23) gives

$$\begin{array}{c} S_{\text{UED}}^{1\text{mol}}(q,T)\propto \frac{1}{q^{4}}ℜ(S_{\text{UED}}^{1\text{mol},\text{elec}}(q,T)+S_{\text{UED}}^{1\text{mol},\text{nuc}}(q,T)\\ +\;S_{\text{UED}}^{1\text{mol},\text{mixed}}(q,T)), \end{array}$$ (25)

where each contribution can be read from Fig. 2,

$$\begin{array}{c} S_{\text{UED}}^{1\text{mol},\text{elec}}(q,T)=\langle \chi_{g}(T)|\sigma_{gg}^{E}(-q)\sigma_{gg}^{E}(q)|\chi_{g}(T)\rangle \\ +\;\langle \chi_{e}(T)|\sigma_{ee}^{E}(-q)\sigma_{ee}^{E}(q)|\chi_{e}(T)\rangle \\ +\;\langle \chi_{g}(T)|\sigma_{eg}^{E}(-q)\sigma_{eg}^{E}(q)|\chi_{g}(T)\rangle \\ +\;\langle \chi_{e}(T)|\sigma_{ge}^{E}(-q)\sigma_{ge}^{E}(q)|\chi_{e}(T)\rangle \\ +\;2ℜ\langle \chi_{g}(T)|\sigma_{eg}^{E}(-q)\sigma_{ee}^{E}(q)|\chi_{e}(T)\rangle \\ +\;2ℜ\langle \chi_{e}(T)|\sigma_{ge}^{E}(-q)\sigma_{gg}^{E}(q)|\chi_{g}(T)\rangle \end{array},$$ (26)

$$\begin{array}{c} S_{\text{UED}}^{1\text{mol},\text{nuc}}(q,T)=\langle \chi_{g}(T)|\sigma_{gg}^{N}(-q)\sigma_{gg}^{N}(q)|\chi_{g}(T)\rangle \\ +\langle \chi_{e}(T)|\sigma_{ee}^{N}(-q)\sigma_{ee}^{N}(q)|\chi_{e}(T)\rangle \end{array},$$ (27)

$$\begin{array}{c} S_{\text{UED}}^{1\text{mol},\text{mixed}}(q,T)=\langle \chi_{g}(T)|\sigma_{gg}^{E}(-q)\sigma_{gg}^{N}(q)|\chi_{g}(T)\rangle \\ +\;\langle \chi_{e}(T)|\sigma_{ee}^{E}(-q)\sigma_{ee}^{N}(q)|\chi_{e}(T)\rangle \\ +\;2ℜ\langle \chi_{g}(T)|\sigma_{eg}^{E}(-q)\sigma_{ee}^{N}(q)|\chi_{e}(T)\rangle \\ +\;2ℜ\langle \chi_{e}(T)|\sigma_{ge}^{E}(-q)\sigma_{gg}^{N}(q)|\chi_{g}(T)\rangle . \end{array}$$ (28)

## VI. FREQUENCY-RESOLVED XRD

If the scattered light is frequency-dispersed, it is possible to separate the elastic and the inelastic (Stokes and anti-Stokes) contributions to the signal. We have three contributions

$$\begin{array}{c} S_{\text{XRD}}^{\text{elastic}}(q,T,\omega_{s}=\omega_{x})\propto ℜ\langle \chi_{g}(T)|\sigma_{gg}^{E}(-q)\sigma_{gg}^{E}(q)|\chi_{g}(T)\rangle \\ +\;\langle \chi_{e}(T)|\sigma_{ee}^{E}(-q)\sigma_{ee}^{E}(q)|\chi_{e}(T)\rangle ) \end{array},$$ (29)

$$S_{\text{XRD}}^{\text{Stokes}}(q,T,\omega_{s}=\omega_{x}-\omega_{eg})\propto ℜ(\langle \chi_{g}(T)|\sigma_{eg}^{E}(-q)\sigma_{eg}^{E}(q)|\chi_{g}(T)\rangle ),$$ (30)

$$S_{\text{XRD}}^{\text{anti}-\text{Stokes}}(q,T,\omega_{s}=\omega_{x}+\omega_{eg})\propto ℜ(\langle \chi_{e}(T)|\sigma_{ge}^{E}(-q)\sigma_{ge}^{E}(q)|\chi_{e}(T)\rangle ).$$ (31)

**FIG. 4.**
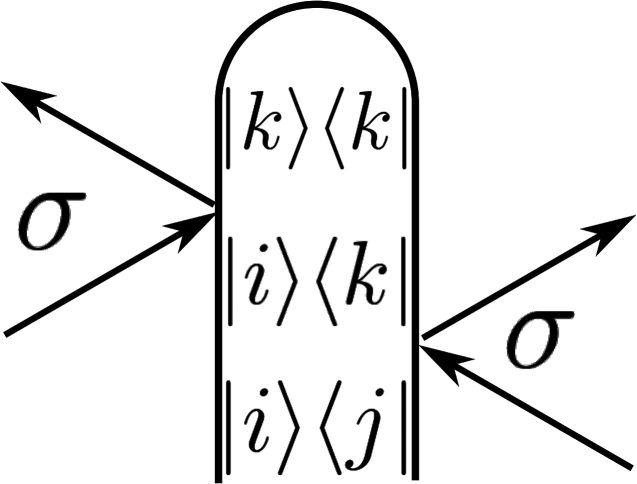
Loop diagram for frequency-dispersed x-ray diffraction, Eq. (32).

Without frequency resolution, one recovers Eq. (14). Equations (29)–(31) assume that one can separate the contributions. In realistic cases, one has to compute the contribution from the different states within the bandwidth of the pulses, see the diagram in Fig. 4,

$$\begin{array}{c} S_{\text{XRD}}(q,T,\Delta \omega )\propto ℜ\sum_{\mathrm{ijk}}\int d\omega_{x}\langle \chi_{i}(T)|\sigma_{ik}^{E}(-q)\frac{a_{X}^{2}(\omega_{x})}{\Delta \omega -{\hat{\omega }}_{ik}+i\varepsilon }\sigma_{kj}^{E}(q)|\chi_{j}(T)\rangle \end{array},$$ (32)

where $a_{X}(\omega_{x})$ is the incident x-ray pulse envelope, $\Delta \omega =\omega_{s}-\omega_{x}$ and ${\hat{\omega }}_{ij}$ is the transition frequency between states *i* and *j*. Importantly, ${\hat{\omega }}_{ij}$ is an operator in nuclear space and contains inelastic vibrational and vibronic contributions to the inelastic scattering. Thus, the resonant factor is not factorized out of the expectation value since it also needs to be averaged over the nuclear wavepacket. Equation (32) is derived in Appendix B.

In order to extract the contribution including nuclear overlaps,

$$2ℜ\langle \chi_{g}(T)|\sigma_{eg}^{E}(-q)\sigma_{ee}^{E}(q)|\chi_{e}(T)\rangle +2ℜ\langle \chi_{e}(T)|\sigma_{ge}^{E}(-q)\sigma_{gg}^{E}(q)|\chi_{g}(T)\rangle ,$$ (33)

one can detect the frequency-integrated signal, Eq. (14), that contains all contributions and subtract from it the ones measured with frequency-resolution, Eqs. (29)–(31).

## VII. CONCLUSIONS

We have presented a rigorous formulation of ultrafast XRD and UED signals suitable for *ab initio* simulations. The main expressions are Eqs. (12), (14), and (16), (17) for the single molecule and the coherent two molecules signals, respectively. We have paid special attention to terms that are usually ignored in diffraction simulations arising from a superposition of states.

Contributions that mix elastic and inelastic scattering require a broadband detector. They usually make only a minor correction to the signal, which explains why they have not been studied so far. Targeted efforts are needed to extract them from the data. Subtraction of the elastic and inelastic diffraction will be measured separately with narrowband detection from the nonfrequency-resolved signal that could single out these terms. Alternatively, correlation measurements using stochastic light also have the potential to separate the various contributions.^3^ These terms involve nuclear wavepackets in different electronic states and are thus highly sensitive to vibronic coherences. They can offer a novel window for conical intersections.

## Data Availability

The data that supports the findings of this study are available within the article.

## APPENDIX A: THE SIGNAL DEFINITION

X-ray diffraction is described by the minimal coupling matter/field interaction Hamiltonian

$$H_{\text{int}}=\frac{e}{2m}\int dr\;\sigma_{E}(r,t)A^{2}(r,t),$$ (A1)

where $\sigma_{E}$ is the molecular electronic charge density operator. The vector potential operator is given by

$$\begin{array}{c} A(r,t)=\sum_{s}\sqrt{\frac{\hbar }{2\epsilon_{0}\omega_{s}\Omega }}(\epsilon_{s}a_{s}e^{i(k_{s}\cdot r-\omega_{s}t)}+\epsilon_{s}^{*}a_{s}^{†}e^{-i(k_{s}\cdot r-\omega_{s}t)})\\ =\sum_{s}A_{s}(r,t)+A_{s}^{†}(r,t). \end{array}$$ (A2)

The x-ray diffraction signal is defined as the integrated rate of photon emission in the detector direction $k_{s}$,

$$S_{\text{XRD}}(k_{s},\Gamma )=\int dt\langle {\dot{N}}_{s}\rangle .$$ (A3)

$\left\langle {\dot{N}}_{s}\right\rangle$ can be calculated using the Heisenberg equation of motion which gives

$$\begin{array}{c} {\dot{N}}_{s}=\frac{i}{\hbar }[H_{\text{int}},N_{s}]\\ =\frac{2}{\hbar }\frac{e}{2m}ℑ\int dr\langle \sigma_{E}(r,t)A_{s}^{†}(r,t)\rangle A_{x}(r,t)\\ =\frac{2}{\hbar }\frac{e}{2m}ℑ\int dr\langle \langle \sigma_{E}(r,t)A_{s}^{†}(r,t)\\ \left|\text{exp}\;(-\frac{i}{\hbar }\int_{t_{0}}^{t}dt\prime \;H_{\text{int},-}(t\prime ))|\rho (t_{0})\rangle \rangle A_{x}(r,t\right) \end{array},$$ (A4)

where $A_{x}(r,t)$ is the classical incoming x-ray field. In the last line, we have explicitly written the interaction picture density matrix propagator, where $H_{\text{int},-}(t\prime )$ is the Liouville space operator version of $H_{\text{int}}(t\prime )$ given in Eq. (A1). Since the scattered photon mode *s* is initially in the vacuum state, this expression vanishes at 0th order in the probe field and a first order expansion must be done to recover the x-ray diffraction signal

$$\begin{array}{c} S_{\text{XRD}}(k_{s},\Gamma )=\frac{2}{\hbar^{2}}{\left(\frac{e}{2m}\right)}^{2}|\epsilon_{x}\cdot \epsilon_{s}{|}^{2}ℜ\int drdr\prime \mathrm{dtdt}\prime \langle \sigma_{E}(r,t)\sigma_{E}(r\prime ,t\prime )\rangle A_{x}(r,t)A_{x}^{*}(r\prime ,t\prime )e^{-i(k_{s}\cdot (r-r^{\prime })-\omega_{s}(t-t^{\prime }))} \end{array}.$$ (A5)

When the field amplitudes $A_{x}(r,t)$ do not depend on the ***r*** (i.e., the incoming fields are plane waves), the integrals over the spatial coordinates correspond to Fourier transforms and we get

$$\begin{array}{c} S_{\text{XRD}}(k_{s},\Gamma )=\frac{2}{\hbar^{2}}{\left(\frac{e}{2m}\right)}^{2}|\epsilon_{x}\cdot \epsilon_{s}{|}^{2}ℜ\int dtdt\prime \\ \langle \sigma_{E}(-q,t)\sigma_{E}(q,t\prime )\rangle A_{x}(t)A_{x}^{*}(t\prime )e^{i\omega_{s}(t-t^{\prime })} \end{array}.$$ (A6)

Finally, assuming that the pulses are impulsive compared to the matter dynamics and incident at a delay *T*, $A_{x}(t)=A_{x}\delta (t-T)$, we can set t=t′=T and recover Eq. (9). Similarly, Eq. (15) can be recovered by assuming that the two-point correlation function in Eq. (A5) can be factorized over molecular pairs.^6^

## APPENDIX B: FREQUENCY-DISPERSED SIGNALS

Without frequency resolution, the signal is given by

$$\begin{array}{c} S_{\text{XRD}}(q,T)\propto ℜ\int d\omega_{s}d\mathrm{tdt}\prime e^{i\omega_{s}(t-t^{\prime })}\langle \sigma^{E}(-q,t)\sigma^{E}(q,t\prime )\rangle A_{x}(t-T)A_{x}^{*}(t\prime -T) \end{array}.$$ (B1)

When the signal is frequency dispersed, the *ω_s_* frequency is resolved and the signal becomes

$$\begin{array}{c} S_{\text{XRD}}(q,T,\omega_{s})\propto ℜ\int dtdt\prime e^{i\omega_{s}(t-t^{\prime })}\langle \sigma^{E}(-q,t)\sigma^{E}(q,t\prime )\rangle A_{x}(t-T)A_{x}^{*}(t\prime -T) \end{array}.$$ (B2)

Using the Fourier transform of the pulse time envelope

$$A_{x}(t-T)=\int \frac{d\omega_{x}}{2\pi }e^{i\omega_{x}(t-T)}a_{x}(\omega_{x}),$$ (B3)

$$A_{x}^{*}(t\prime -T)=\int \frac{d\omega_{x}}{2\pi }e^{i\omega_{x}(t\prime -T)}a_{x}^{*}(\omega_{x}).$$ (B4)

With the change of variable τ=t−t′, the integral becomes

$$\begin{array}{c} S_{\text{XRD}}(q,T,\omega_{s})\propto ℜ\int d\omega_{X}d\omega_{X}^{\prime }\mathrm{dtd}\tau e^{i\omega_{s}\tau }\langle \Psi (t_{0})|G^{†}(t-t_{0})\sigma^{E}(-q)G(\tau )\sigma^{E}(q)G(t-\tau -t_{0})|\Psi (t_{0})\rangle \times a_{x}(\omega_{x})a_{x}^{*}(\omega_{x}^{\prime })e^{i\omega_{x}(t-T)}e^{-i\omega_{x}^{\prime }(t-\tau -T)} \end{array},$$ (B5)

where *G*(*t*) is the molecular propagator that contains the interaction with the actinic pulse. This propagation can be treated numerically in order to include possible non-adiabatic dynamics. Assuming that the pulse is short compared to the dynamics of the molecule, we can make the approximation Ψ(t)=Ψ(t−τ)=Ψ(T). The integral over *t* can then be computed and gives a Dirac delta function $\delta (\omega_{x}-\omega_{x}^{\prime })$,

$$\begin{array}{c} S_{\text{XRD}}(q,T,\omega_{s})\propto ℜ\int d\omega_{x}d\tau e^{i(\omega_{s}-\omega_{x})\tau }\langle \Psi (T)|\sigma^{E}(-q)g(\tau )\sigma^{E}(q)|\Psi (T)\rangle a_{x}(\omega_{x})a_{x}^{*}(\omega_{x}). \end{array}$$ (B6)

The integral over *τ* can be computed assuming that G(τ) is the field-free propagator

$$\int_{0}^{+\infty }d\tau e^{i(\omega_{s}-\omega_{x}+i\epsilon )\tau }e^{-iL_{0}\tau }=\frac{1}{\omega_{s}-\omega_{x}-L_{0}+i\epsilon }.$$ (B7)

Inserting this in Eq. (B6) and expanding over states lead to Eq. (32) in the main text.
